# Hemstitching Men: A Surgical Fantasy

**Published:** 1911-12

**Authors:** 


### Hemstitching Men: A Surgical Fantasy.

We learn from a Minneapolis newspaper that Dr. E. Wyllys Andrews of Chicago has invented a surgical sewing machine. This does away with the handling of the needle and thread by the operator, and facilitates and hastens operation, and thus relieves the patient sooner from the dangers of prolonged ether narcosis.

Unfortunately the item does not add that Dr. Andrews has also devised the necessary apparatus for rolling the patient out to such a flattened condition as will enable him to be deftly passed beneath the needles of the sewing machine. When this feat is accomplished we shall soon see much improvement in this painful portion of the surgical technic. Medical journals will soon teem with articles in which the various merits of hemstitch, cross-stitch and other devices hitherto only known to the fair sex will be presented and hotly debated at medical societies. We shall hear of the peritoneum being puffed, hemmed, felled, gored and otherwise fancifully manipulated, while the great omentum will certainly be the better for artistic shirring. A too enterprising hernial protruison, it may confidently be predicted, will, in that happy era, be restrained by quilting.

But we are getting beyond our depth; and having floundered along thus far, beg leave to submit the remaining discussion of the question to the doctor's wife. Undoubtedly that worthy personage has long been persuaded that her husband's ear would be improved by a band of passementerie, and perhaps some other parts of his anatomy might be the better for judicious trimming. Undoubtedly one effect of the innovation will be greatly to enhance the importance of woman in medicine, for in this department she can certainly give us cards and spades, and beat us out of our boots, if not to a frazzle.—Editorial *The American Journal of Clinical Medicine,* November, 1911.

